# Effects of probiotic supplementation on gut barrier function in combat athletes during pre-competition weight loss

**DOI:** 10.3389/fnut.2026.1857878

**Published:** 2026-07-15

**Authors:** Jing Wang, Mei-yi Lai, Shi-jiao Yang, Zhen-qin Cheng, Wan-ting Shen, Qun Zuo

**Affiliations:** 1 School of Exercise and Health, Shanghai University of Sport, Shanghai, China; 2 School of Athletic Performance, Shanghai University of Sport, Shanghai, China

**Keywords:** combat athletes, gut microbiota, intestinal barrier function, probiotics, weight loss

## Abstract

Combat sports athletes frequently experience gastrointestinal disturbances during pre-competition weight management. This study aimed to investigate the effects of probiotic supplementation on gut barrier function in combat athletes undergoing rapid weight loss prior to competition. Twenty-four elite male athletes were randomized to receive either a complex sports probiotic (1.5 × 10^10^ CFU, twice daily) or placebo for 4 weeks. Results showed that probiotic supplementation significantly reduced gastrointestinal symptoms and gut permeability markers (Zonulin, intestinal fatty acid-binding protein, D-lactate), increased secretory immunoglobulin A (SIgA) levels, and attenuated systemic inflammation compared to placebo. The intervention enriched beneficial butyrate-producing bacteria (*Blautia*, *Latilactobacillus*) while suppressing pathogenic *Streptococcus*. These findings demonstrate that probiotic supplementation serves as a safe, effective nutritional strategy to maintain gut homeostasis during rapid weight loss in combat athletes.

**Clinical trial registration:**

http://www.chictr.org.cn, identifier ChiCTR2400079908.

## 1 Introduction

Boxing, wrestling, judo, taekwondo, and mixed martial arts are all combat sports—characterized by intense physical contact and direct confrontation between competitors (1). To ensure fairness and offset weight-based advantages, a strict weight-class system is universally implemented. Competitors often undertake weight reduction prior to contests to gain advantages in strength and stamina in lower weight classes (2, 3). Common weight-loss strategies include intensified training loads and restricted energy intake. Additionally, reducing fluid intake and increasing dehydration are often used, such as saunas exposure or wearing rubberized suits during training to augment sweat output (1). Weight cutting may confer advantages in strength and physique for athletes, but studies have shown that it can cause serious health issues, including depletion of glycogen stores (4), electrolyte imbalances (5), acute kidney injury (6), and severe cognitive impairment (7, 8). These health risks are now getting more attention in sports medicine. Moreover, current discussions often overlook the gastrointestinal system, even though gut health is fundamental to an athlete’s health and post-competition recovery.

The gastrointestinal tract features the largest surface area exposed to the external environment, playing a crucial role in nutrient digestion, absorption and immune defense. Its impairment can reduce athletic performance, delay recovery and provoke symptoms such as nausea, vomiting, cramps and bloody diarrhea (9). Throughout pre-competition weight cutting, combat sport athletes face dual physiological and psychological stressors. High-intensity training redistributes blood flow toward skeletal muscle and skin, decreasing splanchnic perfusion and predisposing the gut to ischemia–reperfusion injury (10). Concurrently, energy and carbohydrate restriction, in combination with dehydration, disrupt microbiota homeostasis; the marked reduction in fermentable fiber deprives bacteria of their substrate, lowering the abundance of short-chain fatty acid (SCFA)-producing microbial (8). Because butyrate serves as the primary energy source for colonocytes, its decline compromises epithelial proliferation and repair (11). In a recent cross-sectional survey of elite weight-cutting judo athletes, 41% reported gastrointestinal symptoms during rapid weight loss (RWL) (7), indicating that gut dysfunction induced by RWL is a notable health issue in this population. The intestinal barrier comprises the mechanical, chemical, immune and biological barriers, which collectively protect the host from environmental insults and mediate continuous crosstalk with luminal microbiota and intestinal contents (12). When integrity is perturbed, bacteria and antigens translocate into the submucosa, triggering local and systemic immune responses that predispose to functional disorders and inflammation. During the weight-cutting period, combined insults—including intestinal ischemia and hypoxia, heat stress, and nutrient deprivation—synergistically increase intestinal permeability (13), evoke persistent gastrointestinal discomfort and impair post-exercise recovery, thereby compromising both physiological readiness and psychological state before competition.

The gastrointestinal tract harbors approximately 100 trillion microorganisms, forming an exquisitely intricate ecosystem (14). This gut microbiota influences the host from the immune system to the endocrine system through complex metabolic signaling. Probiotics are defined as “live microorganisms that, when administered in adequate amounts, confer a health benefit on the host” (15). They have now secured a critical role in sports supplements. Evidence suggests that for athletes suffering from training-induced gastrointestinal dysfunction, probiotic intervention effectively reinforces the gut barrier and shortens recovery time (16). Probiotics have been linked to several mechanisms that strengthen the intestinal barrier, including inhibition of pathogen colonization through competitive adhesion to the intestinal epithelial surface and occupation of potential attachment sites (17); the production of fermentation products such as acetic acid, propionic acid, and butyric acid, which can enhance the expression of tight junction proteins and improve the physical barrier function of epithelial cells (18); and modulation of the intestinal mucosal immune system through increased secretion of secretory immunoglobulin A (SIgA) and induction of anti-inflammatory cytokine production, which can help to mitigate inflammatory responses (19).

Weight cutting places immense stress on gastrointestinal function, but current nutritional protocols lack specific barrier-protecting interventions. Probiotics may serve as a promising intervention, as they modulate the gut microbiota and reinforce intestinal barrier integrity. In this study, combat athletes were monitored while receiving probiotic supplementation during their pre-competition weight loss phase. We hypothesize that probiotics mitigate the negative impact of dietary restriction and training stress, maintain intestinal homeostasis and preserve barrier integrity.

## 2 Materials and methods

### 2.1 Participants

The sample size was calculated using G*Power 3.1.9.7 software with the following parameters: significance level *α* = 0.05, statistical power (1-*β*) = 0.80, and effect size Cohen’s *f* = 0.45 (20). Based on these calculations, a minimum of 12 participants was required.

Inclusion criteria: (1) aged between 18 and 26 years; (2) held a National Level 1 or higher certification; and (3) possessed at least 3 years of systematic combat sport training experience.

Exclusion criteria: (1) a history of chronic gastrointestinal disease or related surgeries; (2) use of medication affecting gut function; or (3) any injury or illness contraindicating the weight-cutting regime.

A total of 24 athletes from contact sports disciplines at the School of Competitive Sports, Shanghai University of Sport, were finally enrolled and randomly assigned to two groups. Baseline characteristics of all participants are summarized in Table 1. This study was approved by the Ethics Committee of Shanghai University of Sport (No. 102772023RT155) and was registered at the Chinese Clinical Trial Registry (No. ChiCTR2400079908). Written informed consent was obtained from all participants in this study.

**Table 1:** Basic characteristics of participants.

Variables	Ee (*n* = 12)	Aa (*n* = 12)	*p*-value
Team (boxing/judo/wrestling)	5/3/4	3/4/5	0.772
Age (years)	21.92 ± 2.02	22.08 ± 2.02	0.842
Training experience (years)	9.00 ± 2.83	9.50 ± 2.43	0.647
Height (cm)	174.50 ± 3.40	175.17 ± 4.02	0.665
Weight (kg)	68.17 ± 6.71	71.53 ± 8.41	0.293
Weight reduction (%)	3.65 ± 2.45	2.91 ± 1.87	0.419

### 2.2 Experimental protocol

Throughout the 4-week pre-competition period, athletes in the probiotic group received two sachets of a multi-strain sports probiotic once daily, 1 h after lunch. Each sachet contained a 2-g dose (≥ 1.5 × 10^10^ CFU) comprising *Lactobacillus rhamnosus* R0011, *Lactobacillus swissi Lafti* L10, *Lactobacillus salivarius* HA-118, *Lactobacillus plantarum* Lp-G18, *Lactobacillus swissi* R0052, *Bifidobacterium animalis Lafti* B94, and *Bacillus coagulans* GBI-306086. Strains were selected for their documented effects on intestinal barrier function and immune modulation, their synergistic potential, and their established safety profile in athletic populations. The control group received a placebo identical to the intervention in appearance, taste, packaging, and specifications but devoid of active probiotics, administered in the exact same manner. To ensure maximum efficacy, the probiotic beverages were reconstituted with water at ≤ 40 °C to preserve bacterial viability.

**Figure 1:**
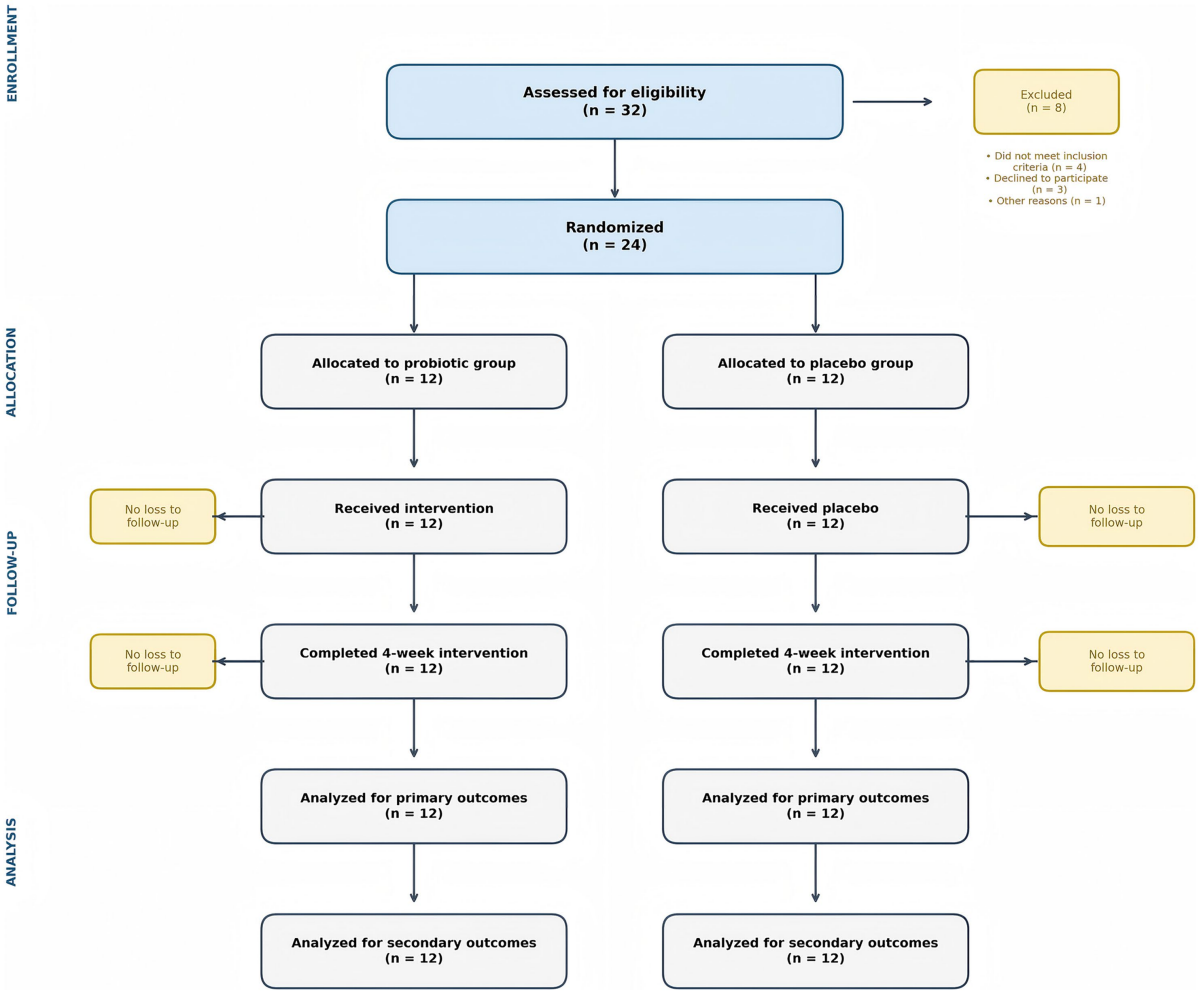
CONSORT flow diagram of participant enrollment, randomization, and follow-up.

This study employed a stratified block randomization design based on combat discipline, weight class, and intended weight-loss magnitude (Figure 1). Participants were allocated to the probiotic or placebo group using computer-generated sequences concealed in sequentially numbered, opaque envelopes. The study utilized a double-blind design with preparations identical in appearance and taste; unblinding occurred only in emergencies. Compliance was monitored via daily sachet returns. Although participants with <80% compliance were eligible for exclusion from the per-protocol analysis, all participants achieved >95% compliance, and none was excluded. During the experiment period, all participants consumed an energy-restricted protocol with a stable daily nutritional structure at a designated athlete canteen. To ensure adherence, participants documented all meals via photographs during the weight-control phase. These records were reviewed by research staff for compliance monitoring and to deliver personalized dietary guidance. Training schedules were standardized to 6 days per week with Sunday off, ensuring consistent duration and content across disciplines. Sessions comprised foundational strength work, sport-specific technical drills and live combat practice.

### 2.3 Measured indicators

This study followed a repeated-measures approach, comparing gastrointestinal (GI) symptoms, intestinal barrier biomarkers, and gut microbiota before versus after the 4 weeks of weight control.

#### 2.3.1 Gastrointestinal symptom assessment

Gastrointestinal Symptom Rating Scale (GSRS) was utilized to evaluate gastrointestinal symptoms. This scale has been proven effective in previous studies on athletes (2). It breaks down GI symptoms into 15 items, covering reflux, indigestion, abdominal pain, constipation and diarrhea. The questionnaire was completed in a quiet, distraction-free environment under the guidance of experimenters. Each item was scored on a seven-point Likert scale (1 = no symptoms, 7 = very severe), yielding a total score of 15–105, with higher values indicating greater symptom severity.

#### 2.3.2 Intestinal barrier biomarkers

Blood samples were collected from the antecubital vein after an overnight fast in the morning. Hematocrit (HCT) were assessed using Sysmex XS-500i hematology analyzer (Sysmex Corporation, Japan). After sampling, the samples were placed at room temperature and protected from light for 30 min. Then, they were centrifuged at 3,500 rpm for 15 min to separate the serum. Next, the supernatant was aspirated and aliquoted into 200-μL sterile cryovials. Finally, samples were immediately transferred to a − 80 °C freezer for storage. Fresh fecal samples were collected using sterile disposable cups. Approximately 5 grams of material was carefully scooped from the center of each sample using a sterile spoon, transferred to pre-labeled sterile cryovials, and sealed for storage at −80 °C. To assess the intestinal barrier function, we used enzyme-linked immunosorbent assay (ELISA), following the standardized operating procedures of each kit strictly. All reagents were procured from specific suppliers. Test kits for intestinal fatty acid-binding protein (I-FABP), lipopolysaccharide (LPS), tumor necrosis factor-alpha (TNF-α) and interleukin-6 (IL-6) were provided by Aibotai Biotechnology (Wuhan), while test kits for SIgA, zonulin, and D-lactate (D-LA) were provided by Enzyme-Linked Bio (Shanghai). Biotree (Shanghai) performed the fecal calprotectin test. Finally, this test was conducted using the SpectraMax^®^ ABS Plus microplate reader from Thermo Fisher Scientific (USA) and the MULTISKAN Sky microplate reader from Thermo Fisher Scientific (USA).

#### 2.3.3 Gut microbiota profiling

Fecal samples (0.25–0.5 g) were weighed into centrifuge tubes, followed by addition of Buffer SA, Buffer SC, grinding beads, and 10 μL RNase A (TIANGEN) to eliminate RNA interference. After thorough homogenization, samples were incubated at 70 °C for 15 min (or at 95 °C for Gram-positive bacteria) to ensure complete cell lysis. Following centrifugation at 12,000 rpm for 1 min, the supernatant was collected. Buffer SH was subsequently added, mixed thoroughly, and incubated at 4 °C for 10 min for protein precipitation. After another centrifugation, the resulting supernatant was transferred to a new tube containing Buffer GFA and Magnetic Bead Suspension G. The mixture was vortexed for 5 min to facilitate specific binding of nucleic acids to the magnetic beads. The beads were then separated using a magnetic stand and washed sequentially with Protein Removal Solution RD and Washing Solution PWD (containing anhydrous ethanol) to remove residual impurities and salt ions. After air-drying at room temperature for 5–10 min, DNA was eluted by adding 50–100 μL of Buffer TB and incubating at 56 °C for 5 min. The supernatant containing high-purity fecal genomic DNA was collected following magnetic separation and stored at −80 °C until further analysis.

The nucleic acid concentration was measured using a Synergy HTX microplate reader from Gene Company Limited (Hong Kong). PCR amplification was performed using full-length 16S rRNA primers, and the integrity of the products was confirmed by 1.8% agarose gel electrophoresis. The PacBio SMRTbell^®^ Prep Kit 3.0 was used to construct the library, and damage repair, end repair, and adapter binding steps were performed. The library was then purified using AMPure^®^ PB magnetic beads. Next, the primers and polymerase were added to the library using the Revio™ Polymerase Kit, and purification was performed using cleanup beads. Finally, the prepared library was sequenced on the PacBio Revio platform.

Bioinformatics and diversity analysis Raw reads were processed with SMRT Link v12.0 software (PacBio, USA) to generate circular consensus sequences (CCS). Barcode demultiplexing and primer trimming were performed, and chimaeras were removed with VSEARCH v2.22.1. Operational taxonomic units (OTUs) were clustered at 97% similarity and taxonomically assigned using the QIIME2 v2022.11 pipeline. *α*- and *β*-diversity indices, phylum- and genus-level community profiles, and between-group statistical tests were computed within the QIIME2 environment.

#### 2.3.4 Body weight and composition monitoring

Body weight and composition were measured using a multi-frequency bioelectrical impedance analyzer (InBody H20, Biospace, South Korea) in the morning following an overnight fast and voiding of bowels and bladder. During the test, participants stood barefoot on the foot electrodes, wore light clothing, and held the electrode handles with both hands. They remained motionless for approximately 2 min. Two measurements were taken to determine weight, body fat percentage, muscle mass, and visceral fat value, with the average used for analysis.

### 2.4 Statistical analysis

All the data results are presented in the form of mean ± standard deviation. Normality was evaluated with the Shapiro–Wilk test. A two-factor repeated measures ANOVA (2 × 2; group × time) was used to analyze outcomes. Simple effects analysis was conducted for interaction effects that were significant. Between-group differences were assessed by independent samples *t*-test or Mann–Whitney *U* test according to normality. Additionally, Spearman’s correlation analysis was performed, with *p* < 0.05 indicating statistically significant differences. The GSRS total score served as the primary outcome for sample size estimation; all other outcomes were secondary or exploratory. We applied Benjamini–Hochberg correction to control the false discovery rate across all outcomes, defining statistical significance at a *q*-value < 0.05. Statistical graphs were generated with GraphPad Prism 10.1.2 and Origin 2024.

## 3 Results

### 3.1 Gastrointestinal symptoms

**Figure 2:**
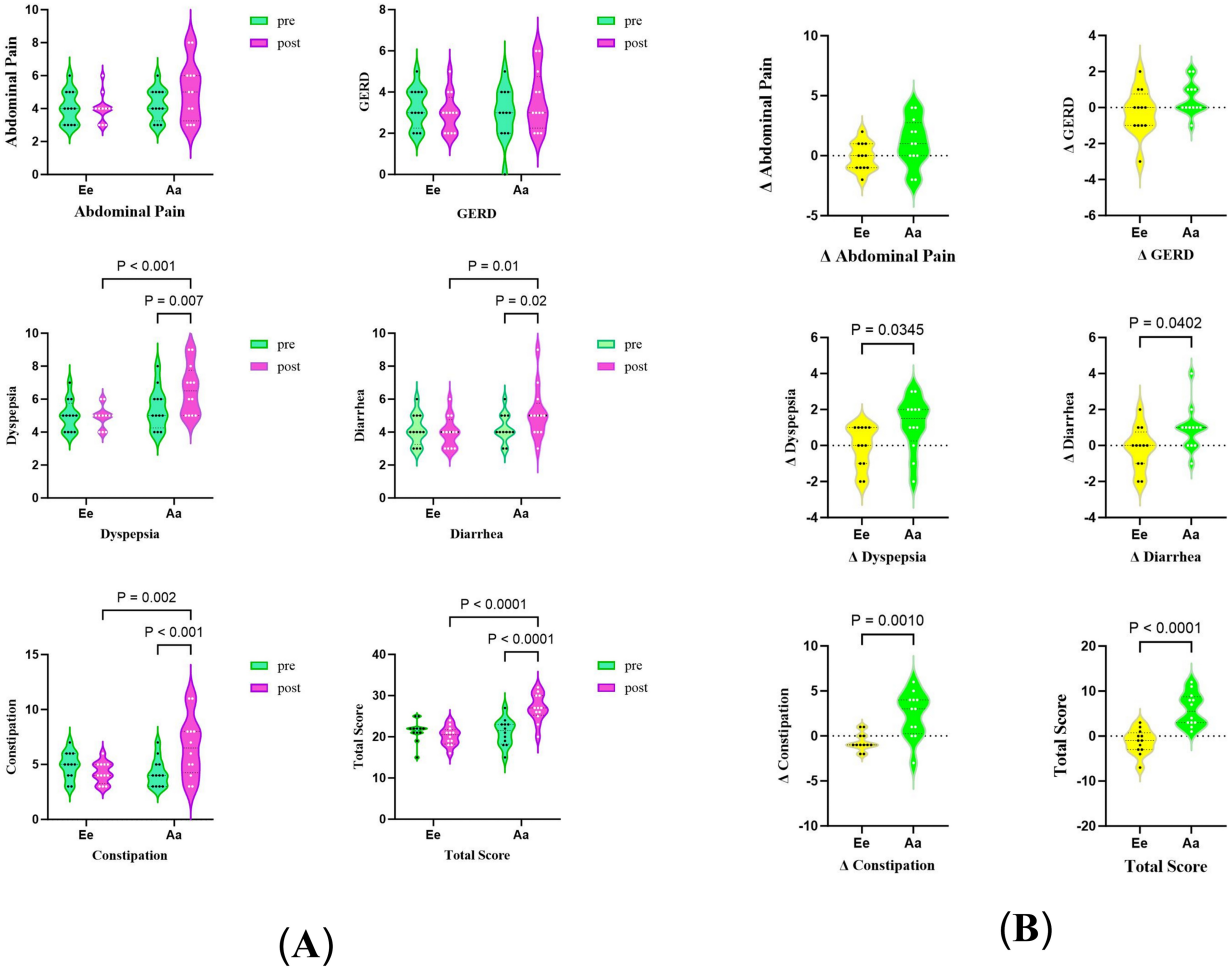
Two-way ANOVA of GSRS scores **(A)** and pairwise difference *t*-test between probiotic and placebo groups **(B)**. GERD, gastroesophageal reflux disease; Ee, probiotic group; Aa, placebo group.

There were time × group interactions for the GSRS total score and for the dyspepsia, diarrhea and constipation subscales (*p* < 0.05; Table 2). Simple-effects analysis showed that these scores increased significantly after weight-cutting in the placebo group and were higher than in the probiotic group. Furthermore, there was a significant group main effect on the score of digestive disorder symptoms. The increment in the dyspepsia, diarrhea and constipation ratings was also significantly greater in the placebo group than in the probiotic group. No significant interactions or main effects were observed for abdominal pain or reflux scores (Figure 2).

**Table 2:** Gastrointestinal symptom rating scale scores of the two groups.

Symptom	Ea (*n* = 12)		Aa (*n* = 12)		Interaction				Main effect of group				Main effect of time			
	Pre-	Post-	Pre-	Post-	*F*	*p*	*q*	η_p_^2^	*F*	*p*	*q*	η_p_^2^	*F*	*p*	*q*	η_p_^2^
Abdominal pain	4.08 ± 0.99	4.00 ± 0.85	4.25 ± 0.97	5.08 ± 1.78	1.960	0.175	0.21	0.082	2.200	0.152	0.206	0.091	1.312	0.264	0.297	0.056
GERD	3.25 ± 0.97	3.00 ± 0.95	2.92 ± 1.31	3.58 ± 1.44	3.836	0.063	0.115	0.148	0.087	0.771	0.771	0.004	0.035	0.383	0.406	0.035
Dyspepsia	5.00 ± 0.95	4.92 ± 0.67	5.42 ± 1.24	6.58 ± 1.51	5.082	0.034^*^	0.115	0.188	7.857	0.010^*^	0.036^#^	0.263	3.817	0.064	0.115	0.148
Diarrhea	4.17 ± 0.94	4.00 ± 0.95	4.25 ± 0.88	5.17 ± 1.59	4.754	0.040^*^	0.103	0.178	2.625	0.119	0.195	0.107	2.279	0.145	0.206	0.094
Constipation	4.92 ± 1.24	4.25 ± 0.97	4.25 ± 1.29	6.58 ± 2.75	14.256	0.001^*^	0.006^#^	0.393	2.115	0.160	0.206	0.088	4.400	0.048^*^	0.108	0.167
Total score	21.42 ± 2.61	20.17 ± 2.29	21.08 ± 3.26	27.00 ± 3.44	28.829	< 0.001^*^	0.005^#^	0.567	28.829	< 0.001^*^	0.005^#^	0.567	12.224	0.002^*^	0.005^#^	0.357

*p < 0.05, ^#^*q* < 0.05. GERD, gastroesophageal reflux disease; Ee, probiotic group; Aa, placebo group.

### 3.2 Intestinal barrier biomarkers

**Figure 3:**
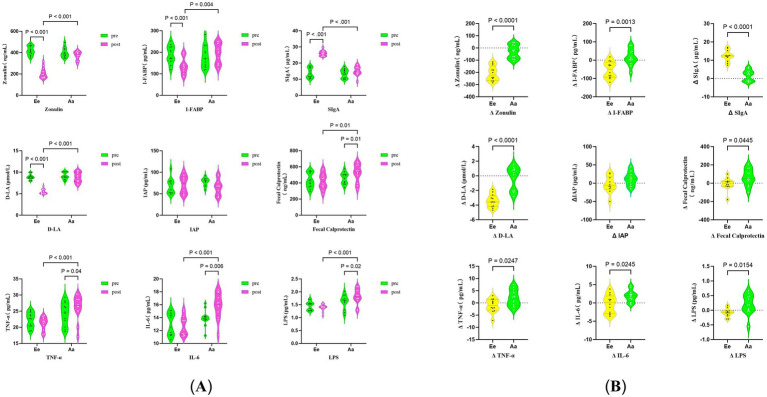
Two-way ANOVA of intestinal barrier biomarkers **(A)** and pairwise difference *t*-test between probiotic and placebo groups **(B)**. I-FABP, intestinal fatty acid-binding protein; SIgA, secretory immunoglobulin A; D-LA, D-lactate; IAP, intestinal alkaline phosphatase; TNF-α, tumor necrosis factor-alpha; IL-6, interleukin-6; LPS, lipopolysaccharide. Ee, probiotic group; Aa, placebo group.

As shown in Table 3 and Figure 3, there were significant interaction effects between time and groups for zonulin, I-FABP, SIgA, D-LA, calcium-binding protein, TNF-α, IL-6, and LPS (*p* < 0.05). However, the interaction effect for calprotectin did not survive FDR correction (*q* > 0.05). Simple-effects analysis indicated that weight cutting in the probiotic group resulted in a significant increase in SIgA and significant decreases in zonulin, I-FABP, and D-LA; these markers differed significantly from those observed in the placebo group (*p* < 0.05). In contrast, the levels of calprotectin, TNF-α, IL-6 and LPS in the placebo group significantly increased and were significantly higher than those in the probiotic group (*p* < 0.05). The probiotic group showed a greater increase in SIgA and larger decreases in zonulin, I-FABP and D-LA; whereas the placebo group showed greater increases in calprotectin and inflammatory markers.

**Table 3:** Intestinal barrier biomarkers of the two groups.

Biomarker (unit)	Ee (*n* = 12)		Aa (*n* = 12)		Interaction				Main effect of group				Main effect of time			
	Pre-	Post-	Pre-	Post-	*F*	*p*	*q*	ηp2	*F*	*p*	*q*	ηp2	*F*	*p*	*q*	ηp2
Zonulin (ng/mL)	418.19 ± 43.44	210.02 ± 50.93	401.31 ± 46.94	371.41 ± 41.29	56.107	<0.001^*^	0.001^#^	0.718	25.104	<0.001^*^	0.001^#^	0.533	100.060	<0.001^*^	0.001^#^	0.820
I-FABP (pg/mL)	189.85 ± 41.28	137.39 ± 39.28	179.72 ± 51.53	194.15 ± 47.39	13.462	0.001^*^	0.002^#^	0.380	2.120	0.160	0.185	0.088	4.348	0.049^*^	0.067	0.165
SIgA (μg/mL)	13.81 ± 3.17	26.09 ± 1.72	13.51 ± 2.74	15.14 ± 2.97	75.358	<0.001^*^	0.001^#^	0.774	37.367	<0.001^*^	0.001^#^	0.629	128.439	<0.001^*^	0.001^#^	0.854
D-LA (μmol/L)	8.89 ± 0.63	5.48 ± 0.60	9.11 ± 0.81	8.79 ± 1.18	35.865	<0.001^*^	0.001^#^	0.673	61.681	<0.001^*^	0.001^#^	0.737	52.054	<0.001^*^	0.001^#^	0.703
IAP (μ/L)	68.21 ± 18.61	71.90 ± 20.52	77.63 ± 9.71	65.54 ± 18.99	2.758	0.114	0.143	0.133	0.782	0.388	0.416	0.042	0.061	0.808	0.808	0.003
Fecal calprotectin (ng/mL)	428.93 ± 92.33	422.35 ± 101.12	459.52 ± 72.06	522.71 ± 112.66	4.540	0.045^*^	0.077	0.171	3.406	0.078	0.104	0.134	2.988	0.098	0.118	0.120
TNF-α (pg/mL)	22.29 ± 2.34	21.19 ± 2.02	24.15 ± 3.59	26.12 ± 3.98	5.816	0.025^*^	0.049^#^	0.209	9.617	0.005^*^	0.020^#^	0.304	0.455	0.507	0.507	0.020
IL-6 (pg/mL)	13.04 ± 1.74	12.82 ± 1.53	13.93 ± 1.53	15.79 ± 2.06	5.898	0.024^*^	0.049^#^	0.211	13.298	0.001^*^	0.006^#^	0.377	3.624	0.070	0.104	0.141
LPS (pg/mL)	1.46 ± 0.17	1.38 ± 0.13	1.61 ± 0.28	1.81 ± 0.30	6.902	0.015^*^	0.045^#^	0.239	13.259	0.001^*^	0.006^#^	0.376	1.198	0.286	0.312	0.052

^*^p < 0.05, ^#^*q* < 0.05. I-FABP, intestinal fatty acid-binding protein; SIgA, secretory immunoglobulin A; D-LA, D-lactate; IAP, intestinal alkaline phosphatase; TNF-α, tumor necrosis factor-alpha; IL-6, interleukin-6; LPS, lipopolysaccharide. Ee, probiotic group; Aa, placebo group.

### 3.3 Hydration status

As shown in Table 4, there were no significant group × time interaction or main effects were observed for either urine specific gravity (USG) or hematocrit (HCT) during the weight control period (all *p* > 0.05, *q* > 0.05), indicating that the athletes maintained a relatively stable hydration status throughout the intervention.

**Table 4:** Hydration status during weight loss period of the two groups.

Biomarker (unit)	Ee (*n* = 12)		Aa (*n* = 12)		Interaction				Main effect of group				Main effect of time			
	Pre-	Post-	Pre-	Post-	*F*	*p*	*q*	η_p_2	*F*	*p*	*q*	η_p_2	*F*	*p*	*q*	η^p^2
USG	1.027 ± 0.003	1.028 ± 0.003	1.028 ± 0.003	1.03 ± 0.001	0.400	0.534	0.641	0.018	2.687	0.115	0.353	0.109	1.600	0.219	0.353	0.068
HCT (%)	44.81 ± 2.25	45.25 ± 2.43	46.30 ± 1.65	45.70 ± 2.11	2.175	0.154	0.353	0.09	1.492	0.235	0.353	0.063	0.050	0.825	0.825	0.002

^*^p < 0.05, ^#^*q* < 0.05. USG, urine specific gravity; HCT, hematocrit. Ee, probiotic group; Aa, placebo group.

### 3.4 Gut microbiota composition and diversity

A total of 490 operational taxonomic units (OTUs) were identified by clustering sequences at 97% similarity across all 44 fecal samples. Venn analysis revealed a core microbiome of 277 OTUs shared among the four groups (A: placebo pre-; a: placebo post-; E: probiotic pre-; and e: probiotic post-). A direct comparison between the post-intervention groups (Ee vs. Aa) showed 411 shared OTUs, with 49 and 30 OTUs unique to the Ee and Aa groups, respectively.

While alpha-diversity indices (Supplementary Figure S1) exhibited an increasing trend after weight cutting, no statistically significant differences were observed between the two groups (*p* > 0.05). In terms of overall community composition, beta-diversity analysis (Supplementary Figure S1) failed to detect any significant divergence or structural segregation between the study arms (*p* > 0.05).

There were significant microbial composition changes at both the genus and species levels. The main bacterial phyla included *Actinobacteria*, *Bacteroidetes*, *Firmicutes*, and *Proteobacteria*. Following weight loss, the *Firmicutes* phylum increased and the *Bacteroidetes* phylum decreased in the probiotic group, whereas the opposite trend was observed in the placebo group. It should be noted that the *Bacteroidetes* abundance declined significantly in the placebo group (*p* < 0.05; Supplementary Figure S2).

At the genus level, the dominant taxa included *Blautia*, *Holdemanella*, *Bifidobacterium*, *Dorea*, and *Streptococcus*. Within-group analysis revealed distinct response patterns (Supplementary Figure S2). In the probiotic group, relative abundances of *Fusicatenibacter*, *Bacteroides*, *Butyricicoccus*, and *Latilactobacillus* increased, whereas *Streptococcus* decreased (*p* < 0.05). In contrast, the placebo group showed a significant increase in *Odoribacter* and significant decreases in *Sutterella* and *Faecalibaculum* (*p* < 0.05).

**Figure 4:**
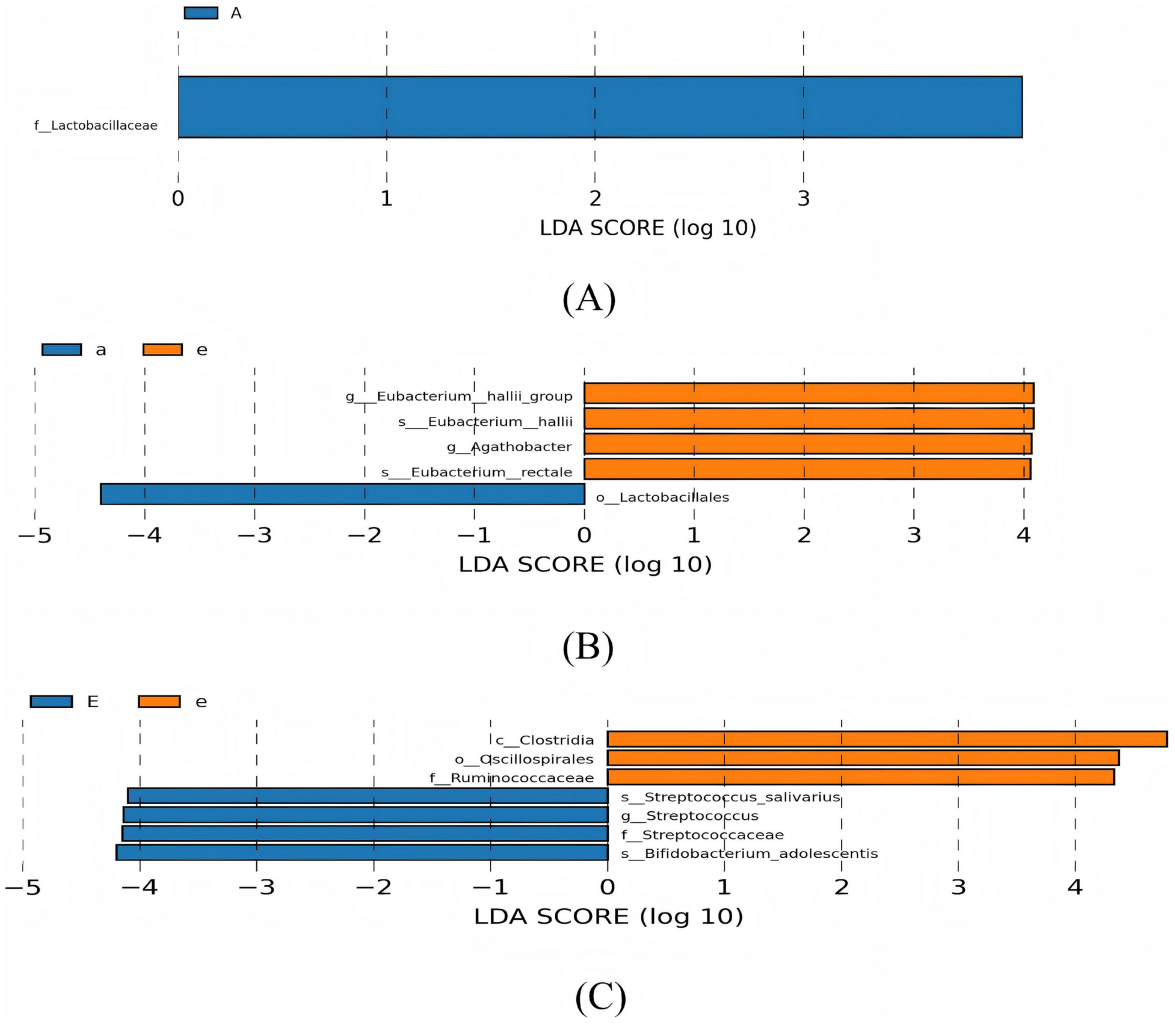
Histogram of linear discriminant analysis (LDA) scores. **(A)** Between-group LEfSe analysis before weight control. **(B)** Between-group LEfSe analysis after weight control. **(C)** Within-probiotic-group LEfSe analysis before and after weight control. The *y*-axis displays the taxa with significant differential abundance between groups, while the *x*-axis presents the logarithmic LDA score for each taxon, with bar lengths intuitively representing the effect size of each discriminative feature. E/e, probiotic group pre−/post-intervention; A/a, placebo group pre−/post-intervention.

Linear Discriminant Analysis Effect Size (LEfSe) analysis, which integrates non-parametric testing with linear discriminant analysis to estimate effect size, was employed to identify microbial taxa that were significantly differentially abundant between groups. The results revealed that the characteristic microbiota in the placebo group before and after weight control were Lactobacillaceae and Lactobacillales, respectively, with no marked within-group shifts in discriminative taxa observed (Figure 4A), suggesting relative stability of the gut microbiota structure in the placebo group. In contrast, compared with the placebo group, the probiotic group exhibited significant enrichment of butyrate-producing bacteria, including *Eubacterium rectale* and Agathobacter, following weight control (Figure 4B). Within-group analysis further demonstrated that anaerobic bacteria, predominantly belonging to Ruminococcaceae, were significantly enriched after weight control in the probiotic group (Figure 4C), indicating that probiotic supplementation facilitated a shift in the characteristic gut microbiota toward fiber metabolism and short-chain fatty acid production.

### 3.5 Correlation between gut microbiota composition and gastrointestinal symptoms

As shown in Supplementary Figure S3, significant correlations were observed between gut taxa and gastrointestinal symptoms. At the phylum level, *Firmicutes* abundance was associated with abdominal pain (*r* = 0.449, *p* < 0.05) and negatively with constipation (*r* = −0.446, *p* < 0.01). In contrast, *Actinobacteria* abundance was inversely related to indigestion (*r* = −0.433, *p* < 0.05) but, positively associated with constipation (*r* = 0.514, *p* < 0.01). *Proteobacteria* abundance showed significant correlation with indigestion scores (*r* = 0.498, *p* < 0.05). At the genus level, *Blautia* abundance correlated negatively with indigestion (*r* = −0.428, *p* < 0.05); *Bifidobacterium* abundance correlated negatively with abdominal pain (*r* = −0.441, *p* < 0.01) and positively with constipation (*r* = 0.487, *p* < 0.01). Notably, *Streptococcus* abundance exhibited a strong positive correlation with diarrhea severity (*r* = 0.622, *p* < 0.01). However, none of these correlations remained significant after FDR correction (*q* > 0.05), suggesting that these associations should be interpreted with caution as exploratory findings requiring validation in larger cohorts.

### 3.6 Correlation between gut microbiota and intestinal barrier biomarkers

Microbiome composition also showed significant correlations with intestinal barrier integrity and systemic inflammation (Supplementary Figure S3). At the phylum level, a negative correlation was observed between the *Firmicutes* phylum and serum D-LA (*r* = −0.378, *p* < 0.01). *Actinobacteria* abundance showed a positive correlation with intestinal mucin (*r* = 0.444, *p* < 0.05), while *Proteobacteria* abundance showed a negative correlation with calmodulin (*r* = −0.445, *p* < 0.05). It should be noted that *Patescibacteria* abundance was negatively correlated with both D-LA (*r* = −0.466, *p* < 0.05) and IL-6 (*r* = −0.556, *p* < 0.01). *Cyanobacteria* abundance was negatively correlated with I-FABP (*r* = −0.436, *p* < 0.05) and calmodulin (*r* = −0.434, *p* < 0.05), but positively correlated with IL-6 (*r* = 0.455, *p* < 0.05). *Holdemanella* abundance showed a negative correlation with SIgA (*r* = −0.439, *p* < 0.05). *Bifidobacterium* abundance showed a positive correlation with zonulin (*r* = 0.423, *p* < 0.05), and *Eubacterium* abundance showed a negative correlation with I-FABP (*r* = −0.427, *p* < 0.05). However, none of these correlations remained significant after FDR correction (*q* > 0.05), indicating that these associations are exploratory and require further validation.

## 4 Discussion

In this study, we found that pre-competition weight loss significantly disrupts gut homeostasis in combat athletes. Probiotic supplementation effectively mitigated the GI symptom and systemic inflammation, enhanced intestinal barrier integrity, and modulated the gut microbiota composition.

### 4.1 Effects of weight control and probiotic supplementation on intestinal barrier function

Gastrointestinal symptoms in athletes during the weight control period were quantified using the GSRS scale to assess clinical manifestations potentially induced by intestinal barrier dysfunction. In our study, while the placebo group exhibited exacerbated gastrointestinal symptoms, the probiotic group showed no significant deterioration from baseline. This suggests that probiotic supplementation may effectively attenuate gastrointestinal distress associated with weight control interventions. As reported by Aitkenhead et al. (21)and Schreiber et al. (20), probiotic supplementation could be a valuable strategy in reducing the incidence of training-related symptoms such as nausea, belching, and vomiting. Intense exercise has been demonstrated to compromise intestinal barrier integrity (21), thereby reducing microbial diversity and promoting the translocation of bacterial endotoxins such as lipopolysaccharides. This triggers systemic inflammation and symptoms like cramps and bloating (22). Furthermore, during the RWL phase, athletes often consume less total energy, carbohydrate, and fat intake, coupled with insufficient dietary fiber intake-factors collectively associated with compromised intestinal barrier integrity (8). Animal studies have demonstrated that starvation induces reversible intestinal atrophy in rats, marked by reduced villus height and consequent impairment of the intestinal physical barrier (23). Research has confirmed that *Escherichia coli strain Nissle* 1917 and *L. rhamnosus GG* contribute to the restoration of intestinal integrity and barrier function (24, 25). I-FABP, a biomarker for epithelial injury located at the tips of intestinal villi (26), typically rises during acute heat stress or dehydration (27). However, the absence of significant I-FABP alterations in the placebo group suggests that the 4-week weight loss phase represents a chronic stressor rather than an acute injury. Conversely, Zonulin, a regulator of tight junctions and marker of permeability (28), was significantly reduced by probiotic intervention in this study. This is consistent with the findings of Lamprecht et al. (29) regarding multi-strain probiotics. Notably, post-intervention Zonulin levels in both groups remained above the upper limit of the healthy reference range (>40 ng/mL) (30), suggesting that elite athletes may experience chronically elevated intestinal permeability due to prolonged training loads. While probiotics mitigate this damage, complete restoration may require longer intervention periods.

Impaired barrier function has been reported to be frequently coupled with inflammatory activation in the placebo group and in exercise stress models (27). This interaction is mechanistically complex but may be attributed to a vicious cycle where energy restriction and high-intensity exercise promote inflammation and permeability. Lipopolysaccharides are involved in metabolic inflammation and have an essential role in the triggering of the TLR4/NF-κB pathway. It has been reported to induce the release of cytokines such as IL-6 and TNF-α, alter gut integrity, and increase fecal calprotectin levels (31). Additionally, it is involved in the systemic immune response and influences the permeability barriers compromised by physical stress. As observed in IBS patients (32), probiotic intervention could be a valuable strategy in enhancing barrier integrity and suppressing inflammation. We think that the decrease in inflammatory markers with probiotic treatment indicates that the supplement has the potential to disrupt the inflammation-permeability cycle in humans; however, the full extent of this protection relative to energy restriction remains to be fully characterized. The increased SIgA levels, particularly apparent in the probiotic group, could either indicate a reinforced immune barrier or an active prevention of pathogen adhesion. We hypothesize that the improvement in barrier function was due to the increase in SIgA secretion, as SIgA can reinforces immune exclusion and prevents pathogen adhesion (33). However, in the absence of significant correlations between SIgA and gut microbiota composition after FDR correction, a direct causal role for SIgA cannot be inferred. The observed enhancement of intestinal barrier function is more plausibly linked to probiotic-induced modulation of butyrate-producing microbiota.

Dehydration is a commonly employed weight-cutting strategy among combat sport athletes. To exclude the potential confounding effects of hydration status on the measured outcomes, we monitored urine specific gravity (USG) and hematocrit (HCT) throughout the study period. The results indicated that the athletes did not experience dehydration or altered hydration status during weight control (Table 2), thereby strengthening the internal validity and reliability of our findings.

### 4.2 Effects of weight control and probiotic supplementation on gut microbiota

In a double-blind randomized controlled trial, Qu et al. (34) demonstrated that two probiotic interventions effectively alleviated GI discomfort in adults, which was attributable to the increased diversity of the gut flora. The abundance of beneficial taxa—including *Bifidobacterium* and *Bacteroides*—increased, whereas that of potential pathobionts such as *Prevotella* and *Sutterella* decreased. These shifts in microbial composition indicate that probiotic intervention enhances intestinal barrier function. Consequently, systemic inflammation is attenuated, and pathogen invasion is blocked, providing the biological rationale for the observed symptom improvement. In our study, the probiotic regimen induced a modest increase in alpha diversity metrics (including Shannon and Chao1), and no broad ecological divergence of Alpha and Beta diversity between the two groups. This absence of substantial structural alteration could be due to the relatively short intervention duration or the capacity of the probiotics to support microbial homeostasis—rather than induce large-scale compositional shifts.

Further compositional analysis at the phylum level revealed a distinct divergence between the groups. The probiotic group was defined by *Firmicutes* enrichment, contrasting with the increased Bacteroidetes and reduced Patescibacteria observed in placebo group. This dominance of *Firmicutes* is functionally significant: by generating butyrate, they stimulate the upregulation of tight junction proteins (e.g., occludin, claudin-1, ZO-1), thereby reinforcing intestinal barrier integrity and attenuating inflammation (32, 35). In contrast, the expansion of *Bacteroidetes* observed in control group is consistently associated with increased intestinal permeability and low-grade systemic inflammation (36). Ni et al. (37) demonstrated that in inflammatory models, elevated *Bacteroidetes* abundance exacerbated tissue injury; conversely, depletion of this phylum restored MUC2 expression and promoted intestinal barrier repair. Collectively, these parallels suggest that probiotic supplementation mitigated weight-loss-associated dysbiosis by preserving a microbial community structure conducive to intestinal barrier integrity.

At the genus level, we observed a marked rise in butyrate producers, notably *Blautia* and *Butyricicoccus*. This shift appears to serve as the basis for intestinal barrier protection, which is consistent with existing literature. In a mouse model of antibiotic-associated diarrhea, Du et al. (38) found that *Bifidobacterium animals* successfully reversed gut dysbiosis. Specifically, the treatment restored microbial balance by enriching beneficial genera—like *Bifidobacterium* and *Akkermansia*—while reducing pathogen loads (*Enterococcus*, *Parasutterella*). Ma et al. (39) observed a similar trend in colitis mice, where *L. salivarius* suppressed *Streptococcus* overgrowth and effectively restored intestinal microbial homeostasis. Our correlation analysis further highlights the role of *Blautia*: its abundance was significantly and inversely correlated with fecal calprotectin levels, indicating a potential anti-inflammatory role. Mechanistically, Su et al. (40) reported that *Blautia A* contributes to cobalamin biosynthesis and butyrate production, thereby fueling colonic epithelial cells and stabilizing the gut microbial ecosystem. This mechanism is further supported by human clinical evidence, for example, a four-week probiotic supplementation not only reduced bloating but also lowered inflammation markers (like calprotectin, D-LA, and LPS) by increasing *Blautia* and *Bifidobacterium* levels (41). Furthermore, increased abundance of *Butyricicoccus* was significantly associated with attenuated CLDN1 upregulation and correlated with reduced disease severity in ulcerative colitis (42). In contrast, the beneficial butyrate-producing genus *Faecalibaculum* decreased in the placebo group, whereas the opportunistic genus *Odoribacter* increased. Collectively, these findings suggest that probiotic supplementation strengthens the intestinal barrier by modulating microbial composition toward anti-inflammatory taxa and away from pathobionts, thereby enhancing both mucosal immunity and epithelial integrity.

Usually, *Bifidobacterium* is regarded as a highly beneficial type of bacteria. It is known for modulating the host immune system and lowering pro-inflammatory cytokines such as TNF-α and IL-6 (43). However, in this study, we observed that the abundances of these bacteria decreased in both groups. The most likely reason for this decline is the energy-restricted, low-carbohydrate diet, especially low-fiber or oligosaccharides intake during weight loss period. Notably, although *Bifidobacterium* abundance was reduced, the probiotic group preserved gut barrier integrity, indicating adaptive modulation of the gut microbiota. Furthermore, the intervention enhanced the abundance of alternative beneficial, butyrate-producing bacteria—including *Blautia*—to compensate for the *Bifidobacterium* deficit. At the same time, probiotic supplementation suppressed opportunistic pathogens such as *Streptococcus*, which may partially offset for the *Bifidobacterium* abundance reduction, because Zhang et al. (44) identified *Streptococcus* as a pathogen associated with recurrent diarrhea in mice.

## 5 Conclusion

Pre-competition weight loss significantly compromises intestinal homeostasis and exacerbates gastrointestinal symptoms in combat athletes. Probiotic supplementation attenuates these adverse effects by modulating gut microbiota composition—specifically enriching butyrate-producing taxa and suppressing pathobionts—and strengthening intestinal barrier integrity, as evidenced by reduced permeability markers (e.g., zonulin, LPS-binding protein) and systemic inflammatory cytokines (e.g., IL-6, TNF-α). These findings support probiotics as a safe, evidence-based nutritional strategy for managing weight loss in combat sports. Future research should optimize strain selection, dosing regimens, and intervention timing, while integrating hydration biomarkers to further elucidate the underlying mechanisms.

## Data Availability

The raw sequencing data presented in this study are deposited in the NCBI Sequence Read Archive (SRA) under BioProject ID PRJNA1484585. The data are publicly accessible at: https://www.ncbi.nlm.nih.gov/bioproject/PRJNA1484585.
