# Supplementary figures and images for: Effects of probiotic supplementation on gut barrier function in combat athletes during pre-competition weight loss
Source: Front Nutr. 2026 Jul 15;13:1857878. doi: 10.3389/fnut.2026.1857878 (PMC13416771; doi:10.3389/fnut.2026.1857878)

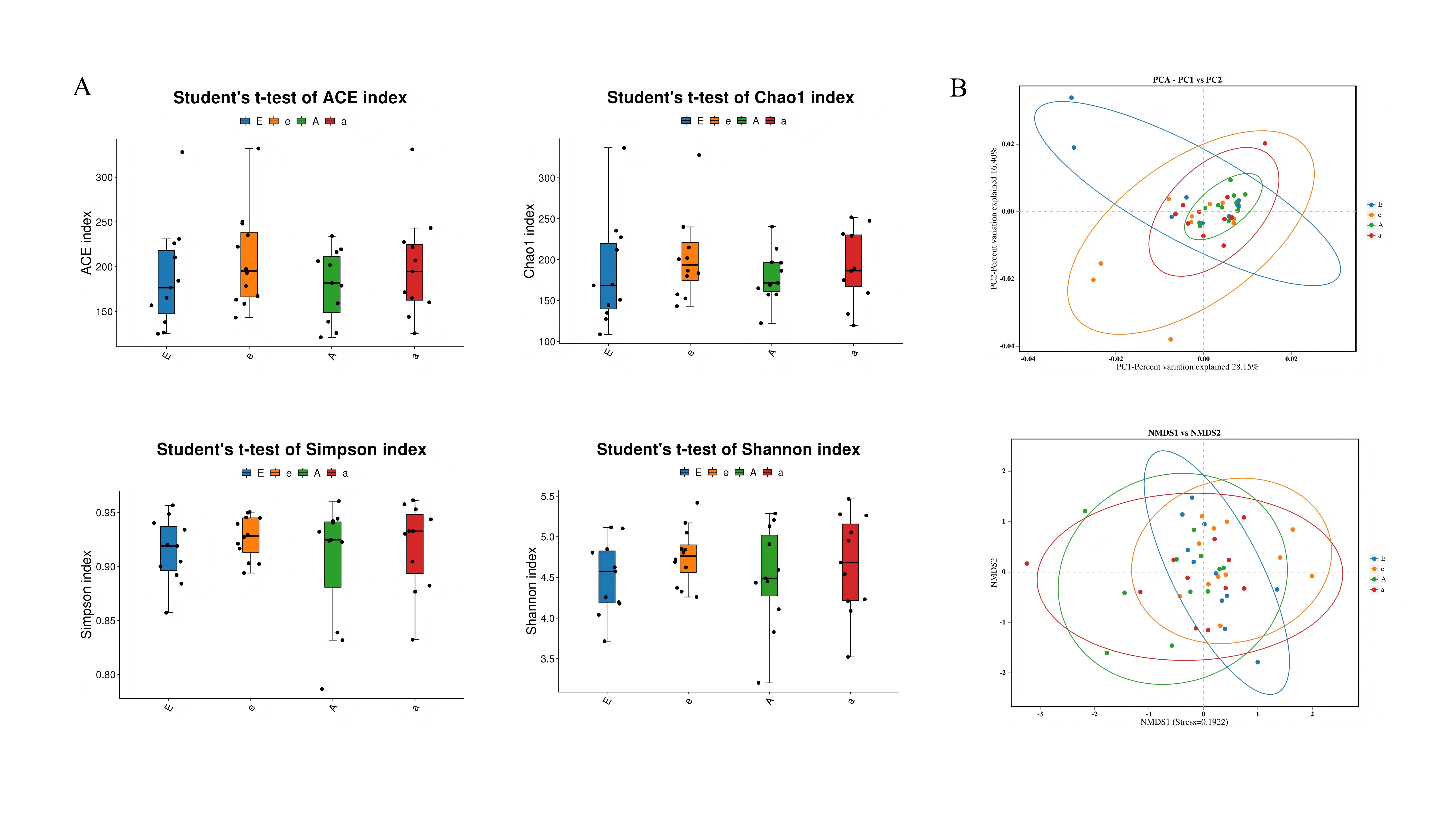

Supplement: Supplementary file 1 [file Image_1.jpeg]

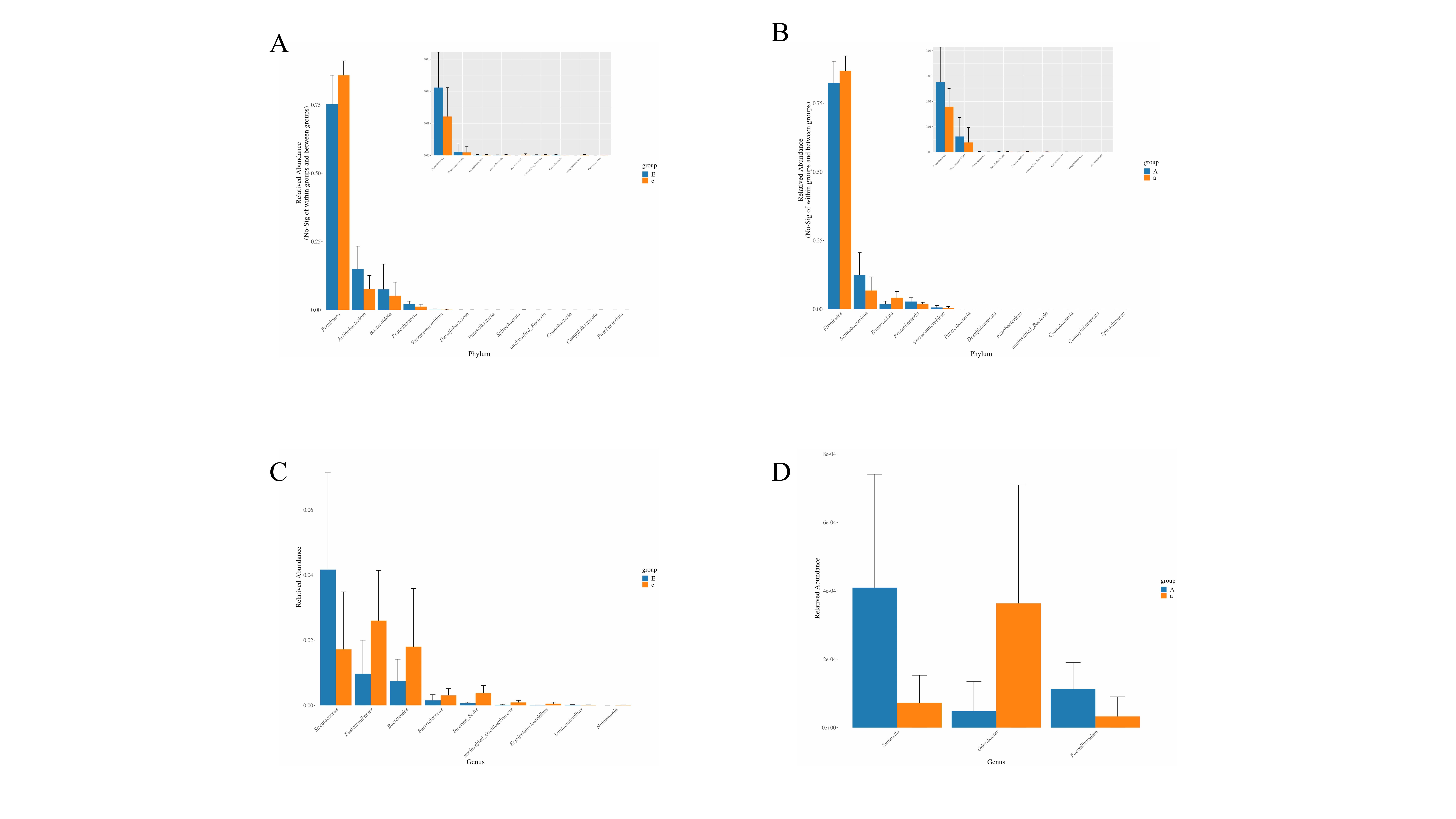

Supplement: Supplementary file 2 [file Image_2.jpeg]

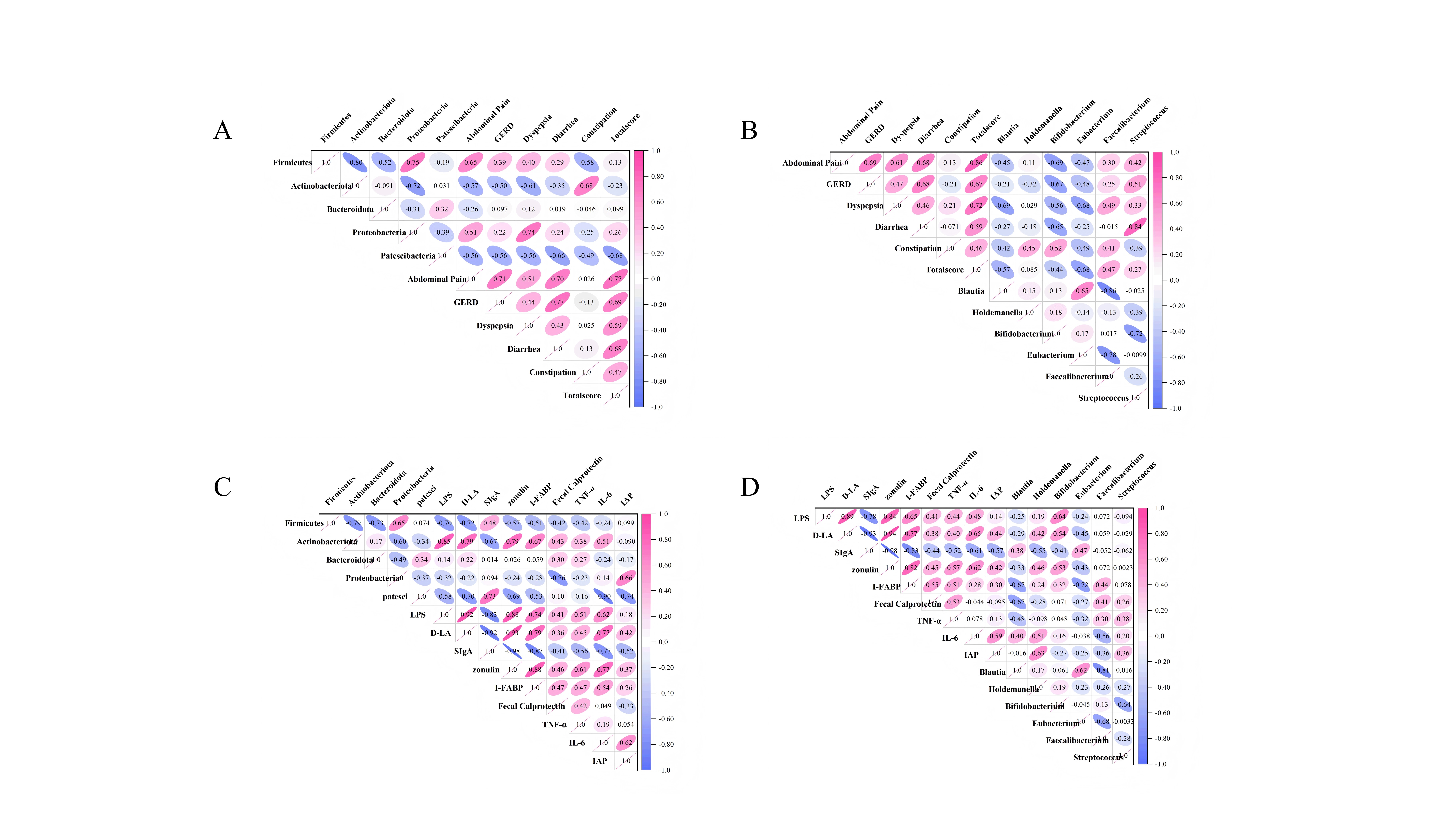

Supplement: Supplementary file 3 [file Image_3.jpeg]
