# Supplementary material for: Effects of probiotic supplementation on gut barrier function in combat athletes during pre-competition weight loss
Source: Front Nutr. 2026 Jul 15;13:1857878. doi: 10.3389/fnut.2026.1857878 (PMC13416771; doi:10.3389/fnut.2026.1857878)
Supplement: Supplementary file 4 [file Supplementary_File_1.docx]

Supplementary Material

# Supplementary Figures and Tables

## Supplementary Figures

**Supplementary Figure 1.** Intergroup difference analysis of intestinal microbiota alpha diversity via Student’s t-test and beta-diversity analysis. PCA, Principal component analysis. NMDS, Non-metric multidimensional scaling. E/e, probiotic group pre-/post-intervention; A/a, placebo group pre-/post-intervention.

**Supplementary Figure 2.** Compositional variation in bacterial communities. (A) Phylum-level bacterial abundance comparison in the Ee group. (B) Phylum-level bacterial abundance comparison in the Aa group. (C) Genus-level bacterial abundance comparison in the Ee group.(D) Genus-level bacterial abundance comparison in the Aa group. E/e, probiotic group pre-/post-intervention; A/a, placebo group pre-/post-intervention.

**Supplementary Figure 3.** Correlation of phylum- and genus-level microbiota with gastrointestinal symptoms and intestinal barrier biomarkers before and after weight control. (A) Correlation of phylum level microbiota with gastrointestinal symptoms.(B) Correlation of genus level microbiota with gastrointestinal symptoms.(C) Correlation of phylum level microbiota with intestinal barrier biomarkers. (D) Correlation of genus level microbiota with intestinal barrier biomarkers. GERD, gastroesophageal reflux disease. I-FABP, intestinal fatty acid-binding protein; SIgA, secretory immunoglobulin A; D-LA, D-lactate; IAP, intestinal alkaline phosphatase; TNF-α, tumor necrosis factor-alpha; IL-6, interleukin-6; LPS, lipopolysaccharide.

## Supplementary Tables

**Supplementary Table 1.**

# Supplementary Table 1. Dietary Intake of the Two Groups

| **Characteristics** | **Ee (n=12)** | | **Aa (n=12)** | | **Interaction** | | | | **Main Effect of Group** | | | | **Main Effect of Time** | | | |
| --- | --- | --- | --- | --- | --- | --- | --- | --- | --- | --- | --- | --- | --- | --- | --- | --- |
|  | **Pre** | **Post** | **Pre** | **Post** | **F** | **P** | **q** | **η_p_2** | **F** | **P** | **q** | **η_p_2** | **F** | **P** | **q** | **η_p_2** |
| Carbohydrate (g) | 307.81  ±45.24 | 185.33  ±28.45 | 277.12  ±50.41 | 179.57  ±38.45 | 1.917 | 0.18 | 0.347 | 0.08 | 1.626 | 0.216 | 0.381 | 0.07 | 149.403 | <0.01^*^ | 0.002^#^ | 0.872 |
| Carbohydrate (g/kg) | 4.56  ±0.77 | 2.86  ±0.53 | 3.89  ±0.62 | 2.59  ±0.49 | 2.702 | 0.114 | 0.311 | 0.109 | 4.471 | 0.046^*^ | 0.153 | 0.169 | 149.515 | <0.01^*^ | 0.002^#^ | 0.872 |
| Protein (g) | 94.79  ±11.99 | 89.76  ±9.09 | 99.15  ±13.15 | 91.76  ±8.77 | 0.237 | 0.631 | 0.757 | 0.011 | 0.723 | 0.404 | 0.582 | 0.032 | 6.593 | 0.018^*^ | 0.068 | 0.231 |
| Protein (g/kg) | 1.39  ±0.16 | 1.36  ±0.13 | 1.4  ±0.22 | 1.34  ±0.22 | 0.31 | 0.583 | 0.729 | 0.014 | 0.033 | 0.857 | 0.863 | 0.002 | 1.439 | 0.243 | 0.405 | 0.061 |
| Fat (g) | 85.95  ±10.95 | 54.05  ±14.09 | 87.87  ±15.65 | 60.43  ±15.33 | 0.655 | 0.427 | 0.582 | 0.029 | 0.672 | 0.421 | 0.582 | 0.030 | 116.005 | <0.01^*^ | 0.002^#^ | 0.841 |
| Fat (g/kg) | 1.27  ±0.21 | 0.83  ±0.22 | 1.25  ±0.28 | 0.86  ±0.23 | 1.021 | 0.323 | 0.510 | 0.044 | 0.03 | 0.863 | 0.863 | 0.001 | 101.165 | <0.01^*^ | 0.002^#^ | 0.821 |
| Calorie (kcal) | 2398.81  ±237.86 | 1599.47  ±219.38 | 2310.88  ±216.88 | 1642.98  ±221.59 | 1.997 | 0.172 | 0.347 | 0.083 | 0.08 | 0.781 | 0.848 | 0.004 | 248.881 | <0.01^*^ | 0.002^#^ | 0.919 |
| Carbohydrate energy  ratio (%) | 51.20  ±4.36 | 46.41  ±4.11 | 47.86  ±6.33 | 43.73  ±6.34 | 0.093 | 0.764 | 0.848 | 0.004 | 2.481 | 0.13 | 0.325 | 0.101 | 16.776 | <0.01^*^ | 0.002^#^ | 0.433 |
| Protein energy ratio  (%) | 32.26  ±2.69 | 30.05  ±4.04 | 34.29  ±5.83 | 32.89  ±5.79 | 0.151 | 0.791 | 0.848 | 0.007 | 2.169 | 0.155 | 0.347 | 0.09 | 3.086 | 0.093 | 0.279 | 0.123 |
| Fat energy ratio  (%) | 15.92  ±2.46 | 22.75  ±3.37 | 17.19  ±1.98 | 22.56  ±2.34 | 1.874 | 0.185 | 0.347 | 0.078 | 0.355 | 0.557 | 0.727 | 0.016 | 129.461 | <0.01^*^ | 0.002^#^ | 0.855 |

Note: ^*^ P < 0.05, ^#^ q < 0.05. Absolute intakes (g) represent total daily amounts; body weight-adjusted intakes (g/kg) are normalized to individual body mass. Energy ratios (%) indicate each macronutrient's proportional contribution to total caloric intake. Ee, probiotic group; Aa, placebo group.
